# Review of "Unfortunate Interference."

**Published:** 1881-11

**Authors:** 


### ARTICLE III.

### *Review of "Unfortunate Interference."*

(Ohio State Journal of Dental Science, August, 1881.)

"Hast thou faith? have it to thyself before God. Happy is he that condemneth not himself in that thing which he alloweth."

"Wad some power the giftie gie us,
To see oursels as ithers see us;
Twad from mony a blunder free us,
And foolish notion."

* * * "Often the pulps of the permanent laterals are drawn out in this way, by their adhering to the roots of the temporary teeth. Not long ago, a popular physician told us he had quite a curiosity to show us and in our line of thought. Taking out a vial he said, 'See here! I took out a tooth for a beautiful little girl the other day, to make room for a new tooth; and look, here is a curious little teat attached to its root.' And he showed us a temporary lateral, with the pulps of the permanent one attached to it. Maimed for life was that beautiful little girl. There is not much excuse for such malpractice. We are not so much surprised that physicians forget, as that they presume to know when so dangerously befogged.

Better far, had he cut off the little girl's finger. Let it be born in mind that a temporary tooth is to be removed only to make room for its own successor. Let it never be extracted on any side issue. When physicians are not posted, they should refer all such cases to dentists."

The complacency with which some presume to teach the depths of wisdom and knowledge upon subjects of which they are as innocent as three months fœtuses, is to say the least remarkable. The easy virtue of would-be-wise men who give credence to the mere babble of pretenders, is matter for animadversion and illumination of the obtuseness with which they jump at conclusions that must conclude.

That an old fogy M. D., should make the mistake of not being able to recognize the product of the inflammatory process set up by the economy to rid itself of a part which had become offensive by having been deprived of normal blood and nerve supply may not be an unheard of blunder, but that an M. D., *and* D. D. S., should forget that the "pyogenic-sac" (in the old purlance) never could include the pulp-gum of a coming permanent incisor tooth, is so far from commendable as to call loudly to him to take a dose of his own prescription and study "the order of appearance in second dentition." It is needful to call to all, who are so careless in study or negligent of the facts of the case in every subject yet dissected and recorded to the fact of the building of each socket of each tooth immediately about the germ-sac in which each tooth is developed; thus making it utterly impossible for any germ to "adhere" to the temporary tooth or root when extracted. Let those who wish to correct "unfortunate interference" not "interfere" when they are *novices* and not *masters*, as proved by the zeal in condemning others, and allowing themselves to rush into fields unknown to them and trodden only by the *very* few, so faithfully as to entitle to deal damnation to those quite as innocent of the special knowledge involved as those these condemn. It is one of the rarities in histological embryology to find a tooth developed without a *bony-cell* or sac in which it is calcified to render it useful as a cutter, teaser or grinder! But that a tooth belonging to the secondary or permanent set has any vascular connections with the temporary or first set, yet remains among the unknown categories of anatomy, physiology and therapeutics—a notable M. D., D D. S., to the contrary notwithstanding.

Let us have less trash and more fact to grace the pages of the "best dental journal extant"—or we shall go on groping as before its advent to illuminate the whole body.

One of the Few.

